# Supplementary figures and images for: Opposing functions of F-BAR proteins in neuronal membrane protrusion, tubule formation, and neurite outgrowth
Source: Life Sci Alliance. 2019 Jun 3;2(3):e201800288. doi: 10.26508/lsa.201800288 (PMC6549137; doi:10.26508/lsa.201800288)

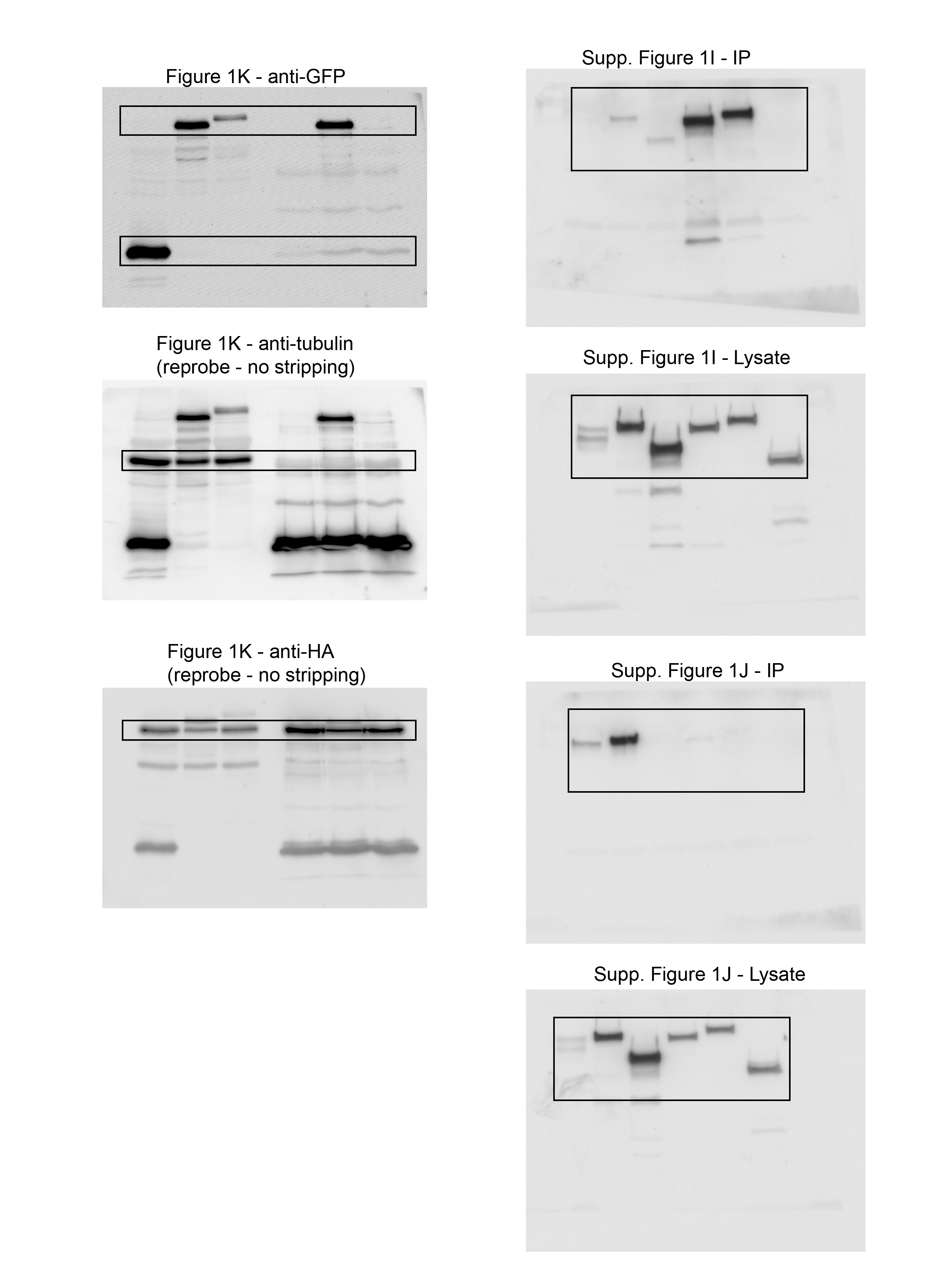

Supplement: Supplementary file 1 [file LSA-2018-00288_SdataF1.jpg]
